# Emergency Department Visits and Disease Burden Attributable to Ambulatory Care Sensitive Conditions in Elderly Adults

**DOI:** 10.1038/s41598-019-40206-4

**Published:** 2019-03-07

**Authors:** Vivian Chia-Rong Hsieh, Meng-Lun Hsieh, Jen-Huai Chiang, Andy Chien, Ming-Shun Hsieh

**Affiliations:** 1 Department of Health Services Administration, China Medical University, Taichung, Taiwan; 2 School of Chinese Medicine, China Medical University, Taichung, Taiwan; 3 Management Office for Health Data, China Medical University Hospital, Taichung, Taiwan; 4 Department of Physical Therapy and Graduate Institute of Rehabilitation Science, China Medical University, Taichung, Taiwan; 5 Institute of Occupational Medicine and Industrial Hygiene, College of Public Health, National Taiwan University, Taipei, Taiwan; 6 Department of Emergency Medicine, Taipei Veterans General Hospital, Taoyuan Branch, Taoyuan, Taiwan; 7 Department of Emergency Medicine, Taipei Veterans General Hospital, Taipei, Taiwan; 8 School of Medicine, National Yang-Ming University, Taipei, Taiwan

## Abstract

Many countries worldwide are aging rapidly, and the complex care needs of older adults generate an unprecedented demand for health services. Common reasons for elderly emergency department (ED) visits frequently involve conditions triggered by preventable infections also known as ambulatory care sensitive conditions (ACSCs). This study aims to describe the trend and the associated disease burden attributable to ACSC-related ED visits made by elderly patients and to characterize their ED use by nursing home residence. We designed a population-based ecological study using administrative data on Taiwan EDs between 2002 and 2013. A total of 563,647 ED visits from individuals aged 65 or over were examined. All elderly ED visits due to ACSCs (tuberculosis, upper respiratory infection, pneumonia, sepsis, cellulitis and urinary tract infection (UTI)) were further identified. Subsequent hospital admissions, related deaths after discharge, total health care costs and disability-adjusted life years (DALYs) were compared among different ACSCs. Prevalence of ACSCs was then assessed between nursing home (NH) residents and non-NH residents. Within the 12-year observation period, we find that there was a steady increase in both the rate of ACSC ED visits and the proportion of elderly with a visit. Overall, pneumonia is the most prevalent among six ACSCs for elderly ED visits (2.10%; 2.06 to 2.14), subsequent hospital admissions (5.77%; 5.59 to 5.94) and associated mortality following admission (17.37%; 16.74 to 18.01). UTI is the second prevalent ACSC consistently across ED visits (2.02%; 1.98 to 2.05), subsequent hospital admissions (2.36%, 2.25 to 2.48) and mortality following admission (10.80%; 10.28 to 11.32). Sepsis ranks third highest in the proportion of hospitalization following ED visit (2.29%; 2.18 to 2.41) and related deaths after hospital discharge (7.39%; 6.95 to 7.83), but it accounts for the highest average total health care expenditure (NT$94,595 ± 120,239; ≈US$3185.02) per case. When examining the likelihood of ACSC-attributable ED use, significantly higher odds were observed in NH residents as compared with non-NH residents for: pneumonia (adjusted odds ratio (aOR): 5.01, 95% confidence interval (CI) 4.50–5.58); UTI (aOR: 4.44, 95% CI 3.97–4.98); sepsis (aOR: 3.54, 95% CI 3.06–4.10); and tuberculosis (aOR: 2.44, 95% CI 1.63–3.65). Here we examined the ACSC-related ED care and found that, among the six ACSCs studied, pneumonia, UTI and sepsis were the leading causes of ED visits, subsequent hospital admissions, related mortality, health care costs and DALYs in Taiwanese NH elderly adults. Our findings suggest that efficient monitoring and reinforcing of quality of care in the residential and community setting might substantially reduce the number of preventable elderly ED visits and alleviate strain on the health care system.

## Introduction

Many populations worldwide are rapidly aging, bringing unprecedented challenges like catastrophic health care expenditure alongside diminishing productivity. Asia has the highest share of elderly people compared to other regions like North America or Europe^1^. In Taiwan, adults aged 65 and older represent 13.9% of the population in 2017 which is a 2.4 percentage point growth from only 4 years ago (11.5%) in 2013^2^. This proportion of elderly population is comparable with others areas in east Asia like Hong Kong and Korea, and is quickly catching up to that of Japan. As a result, the dynamic interplay between Taiwan’s aging demographics and its barrier-free access of the comprehensive health care benefits has generated tremendous pressure on the country’s health system.

Complex care needs of older adults create a great demand for health services. Higher rates of emergency department (ED) visits are typically observed in the elderly compared to other age groups^3,4^. Common reasons for their ED visits include not only age-related chronic problems or fall-related injuries^5^, but they also frequently involve conditions triggered by infections such as pneumonia and urinary tract infections^6^. These conditions can be uniformly classified as ambulatory care sensitive conditions (ACSCs) which can be effectively avoided by sufficient case management and good community care^7,8^. Incidence of tuberculosis, for example, has been proven to decline with quality surveillance and early detection of infection by health care workers in nursing homes (NHs)^9^. As more and more frail older people depend on community and residential care, emerging evidence is indicating that some visits to emergency units are simply preventable and unnecessary due to failed care^7,10^. Consequently, surprisingly high rates of ED visits are made by NH residents^11^. Moreover, these individuals are also more likely to be hospitalized and in need of serious medical attention subsequent to ED visit^12^. As of 2015, approximately 0.8% (23,558 of 2,938,579) of older people aged 65 and over in Taiwan reside in NHs^13^.

Although several studies have acknowledged the fast aging rate globally, none has yet assessed the long-term ED care sought, together with the intensity of resource use and the associated disease burden caused by ACSCs in older adults. The aim of this study is to describe the pattern of elderly ED visits attributable to ACSCs from 2002–2013 and to characterize their disease burden in the 12-year period. We also seek to investigate if NH elderly are at increased risk of acquiring preventable ED visits due to ACSCs using population-wide data.

## Methods

### Study design and setting

The present study adopted a population-based ecological study design. Data were collected for individuals aged 65 years or over between 2002 and 2013. We used the Longitudinal Health Insurance Database 2000 (LHID2000), which is an administrative database that contains original medical claims data of 1 million randomly-sampled beneficiaries drawn in 2000 from the entire population enrolled in Taiwan’s National Health Insurance (NHI) system. Like with a closed-cohort concept, the sampled individuals will remain in the database until end of observation (i.e. 2013 in our study), death, or exit from insurance enrollment. The NHI is the country’s single-payer health care system covering almost 100% of the population for services ranging from ambulatory care to emergency department visits. Patient identification used for data linkage is encrypted and untraceable, so a complete review was waived on the basis of unique identification encryption and the study protocol was approved by the research ethics committee at China Medical University Hospital (CMUH104-REC2-115(CR-2)). The research was performed in accordance with relevant guidelines and regulations.

### Study population

The study sample was restricted to individuals aged 65 or over at the time when they presented at EDs. A total of 563,647 ED visits made by patients aged ≥65 were identified from 2002 to 2013. We examined the sample’s sociodemographic factors including age, sex, area of residence and level of facility visited. Age was categorized into five groups: 65–69, 70–74, 75–79, 80–84 and 85 or older. Residential areas of study subjects were classified according to five geographical areas: north, center, south, east and remote islands. Facility scale was organized into four levels of descending order: medical center, regional hospital, district hospital and local clinic. Status of NH residence in the sample was established based on the status of the patient as indicated on the claims record at the time of his/her ED visit. To investigate the distribution of ACSC-related ED visits according to NH residential status, 1,748 NH residents and 102,284 non-NH residents were identified between 2002 and 2013, respectively. We considered a patient to be a NH resident if his/her status has ever been indicated as a NH resident in the claims history during the study period.

### Measurements

Our goal was to first describe patterns in care and resource use for older people who visited EDs because of ASCSs during the 12-year period. ED visits were first identified on a visit-basis and dates of these visits were used to count the total number of visits for each calendar year. We were then able to determine the total number of patients by attributing all visits made by the same individual. Rate of visits is expressed per 100,000 people and is calculated using the total number of ED visits divided by total number of beneficiaries ≥65 years of age for each year. Similarly, proportion of patient with an ED visit was estimated by dividing total number of elderly patients with a visit by the total number of elderly beneficiaries for each year.

ED visits attributable to ACSCs were identified using diagnostic codes for tuberculosis, upper respiratory infection (URI), pneumonia, sepsis, cellulitis and urinary tract infection (UTI). This classification of the ACSCs adhered to the terminology provided by Purdy *et al*.^14^ and Frick *et al*.^8^, except for sepsis which is a systemic infection that can result from the aforementioned ACSCs. The diagnostic codes were based on International Classification of Diseases, 9th Revision, Clinical Modification (ICD-9-CM): tuberculosis (ICD-9-CM: 010–018), URI (465, 487.1), pneumonia (486, 487.0), sepsis (038, 038.9), cellulitis (528.3) and UTI (599). To minimize misclassification bias, the ACSCs were ascertained only when they appear in the primary diagnosis of the ED record.

Consequences of the ACSC-associated ED visits were also followed. Hospital admissions for subjects admitted to hospital following their ED visit, and related deaths within 2 weeks after their discharge from hospital were studied. We believe that they can represent the severity of condition caused by the infections. To estimate the extent of disease burden generated, total health care costs and disability-adjusted life-years (DALYs) were calculated. The former was the sum of medical expenditure incurred from ED visits and subsequent hospital admissions. The latter were year- and disease-specific estimates retrieved from the Global Burden of Disease Study 2016 results specific to the Taiwanese population and then calculated as an average from 2002 to 2013, irrespective of sex^15^. Unfortunately, the DALY estimate for sepsis was unavailable at the time of study and was not considered in the analyses.

### Statistical analysis

Means, standard deviations and medians were reported for continuous variables, whereas counts, percentages and 95% confidence intervals (CIs) were estimated for categorical variables. Chi-square test or Fisher’s exact test was used to compare the differences in proportions of the variables depending on the nature of distribution. For analysis examining differences in ED use between NH and non-NH elderly, generalized linear modelling was used with adjustment for age, sex and area of residence. Finally, multivariate logistic regression models were used to examine the odds of ED care for ACSCs in NH elderly versus non-NH elderly. Statistical analyses in the present study were performed using SAS 9.4 (SAS Institute, Cary, NC, USA). All p-values were reported using two-side test, and a p-value of <0.05 indicated statistical significance.

## Results

### Temporal trend in elderly ACSC-specific ED visits

**Figure 1:**
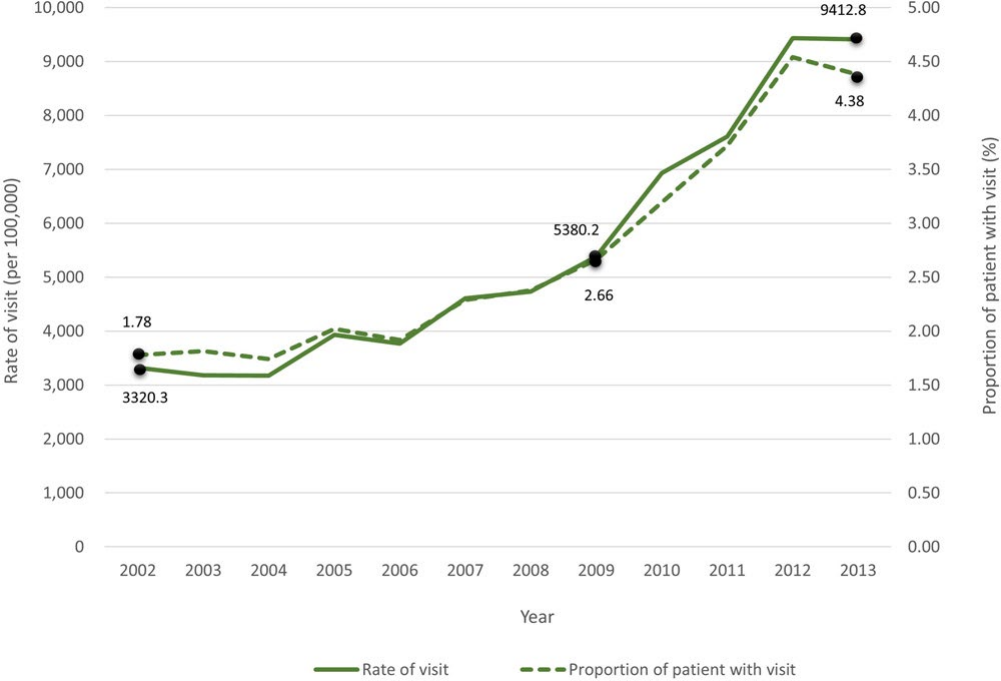
Trend in ACSC-related ED visits among the elderly, 2002–2013 (ACSC – ambulatory care sensitive condition; ED – emergency department).

From 2002 to 2013, there was a consistent rise in the rate of overall ED visits in Taiwanese elderly population, and more than half of ED visits were made by male elderly (53.1%, 95% CI 52.78 to 53.44) (Supplementary Fig. S1 and Table S1). In the same period, we find that there was a steady increase in both the rate of visit and the proportion of elderly with a visit: for years 2002 and 2013, the former was 3320.3 and 9412.8 per 100,000 (2.84-fold increase) and the latter was 1.78% and 4.38% (2.46-fold increase), respectively (Fig. 1). Moreover, the rate of growth (i.e. slope) for both measures accelerated since 2009 (rate of visit: 5380.2 per 100,000; proportion of patient with visit: 2.66%) and attenuated in 2012. Therefore, temporal patterns are analogous for the two measures examining ACSC-specific ED visits, although the degree of increase in the rate of visit seems slightly greater than that of the proportion of elderly with a visit.

### ACSC-related ED disease burden

Table 1 examines the patterns of ACSC-specific ED visits, hospital admissions following ED visit, related mortality after hospitalization, total medical expenditure and DALYs in the elderly population by age group from 2002 to 2013. Of all 563,647 ED visits identified, 66,259 (11.8%) resulted in subsequent hospital admissions, and 13,583 (2.4%) deaths were observed following hospitalization. The probability of subsequent hospitalization appeared to rise with age, from 9.41% (10,980 of 116,663 visits) in 65–69 y to 16.0% (13,370 of 83,603 visits) in >=85 y group. Overall, pneumonia is most prevalent among the six ACSCs for elderly ED visits (2.10%; 2.06 to 2.14), subsequent hospital admissions (5.77%; 5.59 to 5.94) and associated mortality following admission (17.37%; 16.74 to 18.01). Pneumonia-associated DALYs (3,770 ± 4,701) is also the highest, despite its average total health care costs per case (NT$91,166 ± 113,183 or approximately US$3069.56 in 2013; US$1≈NT$29.7) being only second to sepsis (NT$94,595 ± 120,239 or approximately US$3185.02).

**Table 1:** Emergency department (ED) visits, hospital admissions, deaths, total health care expenditure and associated disability-adjusted life years (DALYs) attributable to ambulatory care sensitive conditions in elderly adults, by age group, 2002–2013.

Variable			All			65–69 y			70–74 y			75–79 y			80–84 y			> = 85 y		
			n, mean	%, SD	95% CI, median	n, mean	%, SD	95% CI, median	n, mean	%, SD	95% CI, median	n, mean	%, SD	95% CI, median	n, mean	%, SD	95% CI, median	n, mean	%, SD	95% CI, median
ED visits	All		563647			116663			128963			128602			105816			83603		
	Tuberculosis	5	592	0.11	(0.1–0.11)	81	0.07	(0.05–0.08)	118	0.09	(0.07–0.11)	140	0.11	(0.09–0.13)	133	0.13	(0.1–0.15)	120	0.14	(0.12–0.17)
	Upper respiratory infection	3	7333	1.30	(1.27–1.33)	1799	1.54	(1.47–1.61)	1856	1.44	(1.37–1.5)	1709	1.33	(1.27–1.39)	1230	1.16	(1.1–1.23)	739	0.88	(0.82–0.95)
	Pneumonia	1	11823	2.10	(2.06–2.14)	1334	1.14	(1.08–1.2)	1895	1.47	(1.4–1.54)	2550	1.98	(1.91–2.06)	2824	2.67	(2.57–2.77)	3220	3.85	(3.72–3.98)
	Sepsis	4	4713	0.84	(0.81–0.86)	672	0.58	(0.53–0.62)	946	0.73	(0.69–0.78)	1117	0.87	(0.82–0.92)	993	0.94	(0.88–1.00)	985	1.18	(1.11–1.25)
	Cellulitis	6	114	0.02	(0.02–0.02)	27	0.02	(0.01–0.03)	27	0.02	(0.01–0.03)	31	0.02	(0.02–0.03)	13	0.01	(0.01–0.02)	16	0.02	(0.01–0.03)
	Urinary tract infection	2	11362	2.02	(1.98–2.05)	1885	1.62	(1.54–1.69)	2240	1.74	(1.67–1.81)	2483	1.93	(1.86–2.01)	2357	2.23	(2.14–2.32)	2427	2.90	(2.79–3.02)
Hospital admissions	All		66259			10980			13651			14798			13460			13370		
	Tuberculosis	5	150	0.23	(0.19–0.26)	15	0.14	(0.07–0.21)	24	0.18	(0.11–0.25)	30	0.20	(0.13–0.28)	40	0.30	(0.21–0.39)	41	0.31	(0.21–0.4)
	Upper respiratory infection	4	207	0.31	(0.27–0.35)	35	0.32	(0.21–0.42)	35	0.26	(0.17–0.34)	52	0.35	(0.26–0.45)	42	0.31	(0.22–0.41)	43	0.32	(0.23–0.42)
	Pneumonia	1	3821	5.77	(5.59–5.94)	322	2.93	(2.62–3.25)	501	3.67	(3.35–3.99)	796	5.38	(5.02–5.74)	894	6.64	(6.22–7.06)	1308	9.78	(9.28–10.29)
	Sepsis	3	1520	2.29	(2.18–2.41)	203	1.85	(1.6–2.1)	279	2.04	(1.81–2.28)	316	2.14	(1.9–2.37)	299	2.22	(1.97–2.47)	423	3.16	(2.87–3.46)
	Cellulitis	6	14	0.02	(0.01–0.03)	4	0.04	(0–0.07)	5	0.04	(0–0.07)	2	0.01	(−0.01–0.03)	0	0.00	(0–0)	3	0.02	(0–0.05)
	Urinary tract infection	2	1567	2.36	(2.25–2.48)	202	1.84	(1.59–2.09)	269	1.97	(1.74–2.2)	311	2.10	(1.87–2.33)	362	2.69	(2.42–2.96)	423	3.16	(2.87–3.46)
Deaths	All		13583			2814			2870			3378			2629			1892		
	Tuberculosis	5	162	1.19	(1.01–1.38)	26	0.92	(0.57–1.28)	36	1.25	(0.85–1.66)	51	1.51	(1.1–1.92)	28	1.07	(0.67–1.46)	21	1.11	(0.64–1.58)
	Upper respiratory infection	4	761	5.60	(5.22–5.99)	137	4.87	(4.07–5.66)	196	6.83	(5.91–7.75)	223	6.60	(5.76–7.44)	124	4.72	(3.91–5.53)	81	4.28	(3.37–5.19)
	Pneumonia	1	2360	17.37	(16.74–18.01)	381	13.54	(12.28–14.8)	468	16.31	(14.96–17.66)	602	17.82	(16.53–19.11)	505	19.21	(17.7–20.71)	404	21.35	(19.51–23.2)
	Sepsis	3	1004	7.39	(6.95–7.83)	201	7.14	(6.19–8.09)	220	7.67	(6.69–8.64)	251	7.43	(6.55–8.31)	199	7.57	(6.56–8.58)	133	7.03	(5.88–8.18)
	Cellulitis	6	13	0.10	(0.04–0.15)	3	0.11	(0–0.023)	1	0.03	(−0.03–0.1)	4	0.12	(0–0.23)	5	0.19	(0.02–0.36)	0	0.00	(0–0)
	Urinary tract infection	2	1467	10.80	(10.28–11.32)	240	8.53	(7.5–9.56)	310	10.80	(9.67–11.94)	394	11.66	(10.58–12.75)	314	11.94	(10.7–13.18)	209	11.05	(9.63–12.46)
Total health care costs^a^ (NT$)																				
	Tuberculosis	3	84171	115785	41818	61168	81447	36675	92602	133271	44767	88124	114176	47571	83400	126417	34792	87545	108170	47816
	Upper respiratory infection	6	50110	81322	24711	42363	72539	21526	48440	79691	24803	53221	81937	25506	56392	95487	26664	55912	77169	27372
	Pneumonia	2	91166	113183	47703	81636	109211	42871	85646	111843	42823	94312	120721	47834	93255	116734	49277	94579	105552	54096
	Sepsis	1	94595	120239	49294	88191	142123	43426	91424	121119	45331	97240	114164	54498	96762	115184	51453	97308	113270	53295
	Cellulitis	5	51602	62511	32005	38413	43335	27553	44607	86012	22969	58683	67186	34058	65942	50631	59596	60455	47906	38967
	Urinary tract infection	4	68248	92292	36837	58292	85587	30646	60867	82197	33604	72859	93914	38998	71011	100273	38439	74987	94390	40120
DALYs																				
	Tuberculosis	3	636	840	—	522	707	—	691	904	—	872	1063	—	764	776	—	378	432	—
	Upper respiratory infection	5	47	67	—	93	111	—	77	88	—	57	61	—	39	37	—	18	18	—
	Pneumonia	1	3770	4701	—	2092	2608	—	3042	3625	—	4148	4635	—	4175	4215	—	3249	3508	—
	Sepsis		—	—	—	—	—	—	—	—	—	—	—	—	—	—	—	—	—	—
	Cellulitis	4	57	68	—	42	51	—	53	62	—	66	72	—	60	59	—	45	46	—
	Urinary tract infection	2	1096	1374	—	549	659	—	788	913	—	1074	1168	—	1203	1165	—	1027	1169	—

^a^Total health care costs (per case) = sum of costs from ED visits and subsequent hospital admissions.

ED: emergency department; URI: upper respiratory infection; UTI: urinary tract infection; NT$: New Taiwan Dollar; DALY: disability-adjusted life year; SD: standard deviation.

Similarly, UTI is the second prevalent ACSC consistently across elderly ED visits (2.02%; 1.98 to 2.05), subsequent hospital admissions (2.36%, 2.25 to 2.48) and mortality following admission (10.80%; 10.28 to 11.32). This infection generated an average of 1,096 ± 1,374 DALYs and incurred NT$68,248 ± 92,292 (≈US$2297.91) health spending per case. Sepsis ranks third highest in the proportion of hospitalization following ED visit (2.29%; 2.18 to 2.41) and related death after hospital discharge (7.39%; 6.95 to 7.83), but it accounts for the highest average total health care expenditure (NT$94,595 ± 120,239) per case amongst all ACSCs.

In general, when considering different age groups of elderly, pneumonia remains the most prevalent ACSC in all age groups for ED visits, hospital admissions and associated deaths. The only exceptions are for ED visits in 65–69 y and 70–74 y groups where UTI is most prevalent. Alternatively, sepsis and pneumonia are the highest ranking ACSCs across all age groups in total health care spending and DALYs, respectively – except only when tuberculosis incurred highest spending in the 70–74 y age group.

**Figure 2:**
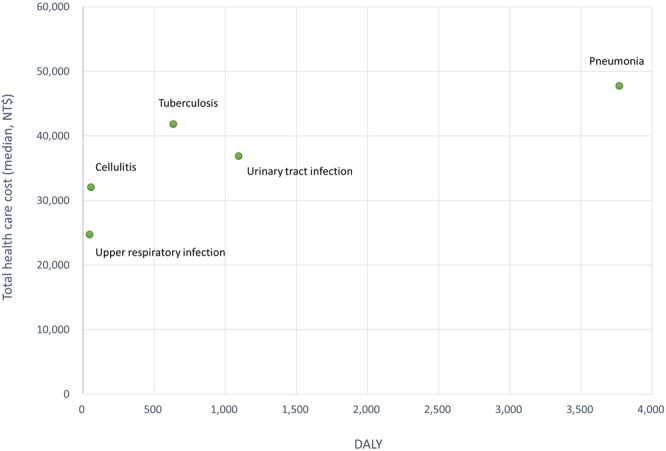
Total health care costs in association with DALYs by ACSC in the elderly (DALY – disability-adjusted life year; NT$ - New Taiwan Dollar).

In Fig. 2, we plotted the association between median total health care expenditure and DALY for each condition examined (except sepsis for which DALY data was unavailable). As expected from above results, pneumonia generated the most substantial disease burden with the highest DALY and highest spending. The observed relationship roughly resembles a straight line with positive direction (i.e. ACSC with higher DALY incurs higher costs). URI and cellulitis contributed to the least level of disease burden.

### Nursing home residence and ACSCs

Finally, we examined differences in the prevalence of ACSCs for elderly adults from and not from NHs in Table 2. Demographics of NH residents including age, sex and area of residence were statistically different from that of non-NH residents: the former is somewhat older in age, consisted of more females and tend to live in the center and south areas of the country. More importantly, a significantly higher percentage of NH residents was identified with pneumonia (30.15% vs. 8.38%; p < 0.0001), UTI (25.23% vs. 7.08%; p < 0.0001), sepsis (12.81% vs. 4.06%; p < 0.0001) and tuberculosis (1.49% vs. 0.64%; p < 0.0001) relative to their non-NH counterparts. After adjusting for age, sex, area of residence and hemodialysis status, our multivariate analyses indicated that NH elderly had significantly higher likelihoods of ED visits owing to ACSCs: pneumonia (adjusted odds ratio (aOR): 5.01, 95% CI 4.50 to 5.58); UTI (aOR: 4.44, 95% CI 3.97 to 4.98); sepsis (aOR: 3.54, 95% CI 3.06 to 4.10); and tuberculosis (aOR: 2.44, 95% CI 1.63 to 3.65) (Table 3). On the contrary, ED utilization due to URI and cellulitis between NH and non-NH residents did not differ.

**Table 2:** Ambulatory care sensitive condition-related ED visits for nursing home and non-nursing home residents, 2002–2013.

Variable		Nursing home elderly				P value*
		No (n = 102,284)		Yes (n = 1,748)		
		N	%	N	%	
Age (year)						<0.0001
	65–69	29,787	29.12	379	21.68	
	70–74	28,394	27.76	441	25.23	
	75–79	20,960	20.49	477	27.29	
	80–84	12,544	12.26	301	17.22	
	> = 85	10,599	10.36	150	8.58	
Sex						<0.0001
	Male	54,859	53.63	841	48.11	
	Female	47,424	46.37	907	51.89	
Area of residence						<0.0001
	North	43,596	43.51	653	38.10	
	Center	18,179	18.15	370	21.59	
	South	33,518	33.46	637	37.16	
	East	3,865	3.86	46	2.68	
	Remote Islands	1,029	1.03	8	0.47	
ACSC	Tuberculosis	653	0.64	26	1.49	<0.0001
	Upper respiratory infection	7,051	6.89	130	7.44	0.3741
	Pneumonia	8,567	8.38	527	30.15	<0.0001
	Sepsis	4,151	4.06	224	12.81	<0.0001
	Cellulitis	82	0.08	1	0.06	0.99^†^
	Urinary tract infection	7,246	7.08	441	25.23	<0.0001

*Chi-square test; ^†^Fisher exact test

ACSC - ambulatory care sensitive condition.

**Table 3:** Multivariate analyses for ED visits by nursing home (vs. non-nursing home) residents due to ACSCs.

ACSC	Nursing home residence (65 years or above)				Nursing home vs. non-nursing home		
	No		Yes		aOR	95% CI	P value
	Event n	Event %	Event n	Event %			
Tuberculosis	653	0.64	26	1.49	**2.44**	(1.63–3.65)	<0.0001
Upper respiratory infection	7051	6.89	130	7.44	**1.15**	(0.96–1.38)	0.1424
Pneumonia	8567	8.38	527	30.15	**5.01**	(4.50–5.58)	<0.0001
Sepsis	4151	4.06	224	12.81	**3.54**	(3.06–4.10)	<0.0001
Cellulitis	82	0.08	1	0.06	**0.81**	(0.11–5.80)	0.8299
Urinary tract infection	7246	7.08	441	25.23	**4.44**	(3.97–4.98)	<0.0001

aOR: adjusted odds ratio (adjusted for age, sex, area of residence and hemodialysis).

CI: confidence interval.

## Discussion

### Main findings

Based on population-based individual-level data from 2002 to 2013, our findings suggest that adverse health and economic consequences result from the rapidly aging demographics, and the accelerated growth in ACSC-related ED patients and visits is evident in the elderly population. Our study also provides evidence that the prevalence of ACSCs is much higher in the NH residents relative to non-NH residents, so are their adjusted likelihoods for using ED care due to ACSCs such as pneumonia, UTI, sepsis and tuberculosis.

### Comparison with previous studies

In spite of the scarcity of recent studies examining longitudinal patterns of elderly ED resource use, our results confirm previous findings which suggest an increasing temporal trend in ACSC-related ED care and disease burden in frail older people. A study of NH residents in the United States (US) from 2001 to 2010 observed a growth of almost three-fold in the overall ED visit rate^7^. The same study also showed that the rate of ED visits due to ACSCs has increased. Our results further indicated that the proportion of patients with ED visit for ACSCs resulting in subsequent hospital admissions increased with their age.

There are several plausible reasons for the progressive increase in ED visits for ACSCs. For factors within the health care system, it can be an indication of poor community and residential care quality. Unfortunately, training capacity for related workforce and the overall infrastructure for elderly care still require much improvement^16,17^. Another factor might be the present reimbursement policies that prohibit prolonged admission stays, generating earlier discharge of patients and increased subsequent ED visits. For causes outside the system, one could be the lack of family support and care from family members as a result of the changing household structure observed in recent years; young adults tend to work away from home, leaving the older “stay-behind” adults living alone at home or even at residential care^17,18^.

Among the ACSCs and infections examined, pneumonia contributes to the highest proportion of ED visits, subsequent hospitalizations, deaths, as well as the magnitude of disease burden. Moreover, it appears to be much more prevalent in NH residents compared to non-NH elderly as previously suggested^19–21^. Analogous results were obtained in a recent US study where pneumonia accounted for majority of ED visits among older people residing in NHs^7^. Predisposing factors to this infection among elderly population could include declined functional capability and immunity and the use of nasogastric tubes^19,22^. A previous Taiwanese study has also showed that residing in NH significantly increases the odds of patients getting an infection^23^.

Additionally, UTI attributes to the second highest proportion of overall ED visits, subsequent hospitalizations, related death and DALYs which is similar to the findings of a previous study conducted using a nationally representative sample in the US^24^. We further found that the prevalence of UTI is substantially higher in NH residents relative to those not in NH care, which is consistent with previous findings^7,21^. This infection is common in older adults living in NHs because of their old age, comorbidities and the use of indwelling urinary catheters^25,26^.

Strategies on the appropriate management and care of patients with sepsis have raised a great deal of awareness, particularly since the updated fourth revision of the Surviving Sepsis Guidelines in 2017^27,28^. Sepsis is another inflammatory condition that was observed with high ED use and mortality among the elderly. Moreover, consistent with earlier findings, we found that older adults from NHs are more likely to have sepsis-related ED visits compared with non-NH residents^21,29^. In this secondary analysis of ED visits in the US, up to 57% of all ED visits were made by people 65 years or older with severe sepsis, and higher mortality rate was associated with NH residence. Other infections in the areas of respiratory system (i.e. upper respiratory system) and skin and soft tissue (i.e. cellulitis) are also common among NH residents^20^. Since the ACSCs examined in this study including pneumonia, UTI, cellulitis and sepsis can be prevented, they are classified as health care-associated infections. In fact, they have been frequently used as indicators for health care quality in countries including the UK and the US^8,30,31^.

Unique from past studies, we further investigated the intensity of health care costs and burden of disease generated by each of the ACSCs. In general, the average monthly income in 2002 and 2013 for the Taiwanese population was NT$41,530 and NT$45,664, respectively^32^. Our analyses showed that an average of NT$94,595 (US$3185.02) is spent per ED-admitted sepsis case, which exceeds twice the monthly average income, despite a major proportion may not come from direct payments. The high expenditure could be partly due to the administration of antibiotics during emergency care. Still, among the ACSCs examined, the lowest mean cost was observed in URI cases (NT$50,110 or US$1687.21 per case) which still exceeds the average income. Therefore, the disease burden caused by elderly ACSC ED care is apparent.

We also detected a direct association between health care costs and DALYs, where ACSCs with high incurred costs also resulted in high burden of disease. This suggests that these common infections can simultaneously generate financial as well as health burden in older adults.

### Strengths and weaknesses of this study

Using a population-based database developed from the single-payer universal health care system, we are able to demonstrate the substantial ACSC-associated economic and health consequences in the elderly, and the significantly increased prevalence of the ACSCs among NH residents. The present study is unlike existing studies where they had relatively shorter observation periods for populations from defined areas^4,7,10,24,33,34^. We had an observation period of 12 years and the data was nationally representative. Nevertheless, this study is subjected to several limitations. First, length of stay for hospitalizations, re-admissions and detailed procedures and medication were not examined to more accurately describe the intensity of resource use due to the ACSCs (i.e. our results may be underestimated). Since we were only concerned with the date of ED visit and not the length of stay, visits made within a single year or across years were handled similarly. Second, the costs were estimated using point values in the medical claim records so they do not represent actual incurred costs. In fact, the real spending may actually be slightly lower after budget allocation process under the current global budget system. Third, we considered sepsis as an ACSC which was not included in the previous terminology^8,14^. However, this can be justified as sepsis is a systemic infection that can result from the ACSCs and it seems relevant to include it in the discussion of appropriate care and performance improvement to reduce mortality due to sepsis^27^. Moreover, emerging evidence is suggesting that is important to consider sepsis as a part of the broadened definition for ACSC, since it is increasingly seen in patients whose conditions could potentially be avoided or treated early^21,35^. Also, for the scope of the study, we only focused on the acute ACSCs and did not explore chronic ACSCs such as asthma and diabetes. Fourth, it is possible that a patient would change NH resident status during the study period. For simplicity, we considered a patient to be a NH resident if he/she has ever been in the NH residence. This is justifiable because our study is ecological in nature. We believe that future studies with better suited designs are warranted to examine the time-dependent attributes of the patients and their causal association with ACSC-related care. Finally, as with all studies using administrative data, there is a possibility for misclassification of ACSC-related ED visits from errors in the diagnostic codes. Thus, to overcome this shortcoming, we only considered ED visits that listed the studied ACSCs in the primary diagnosis.

### Meaning of the study

While the goal of this study was not to compare the level of influence on ED utilization by the ACSCs relative to the other factors, we acknowledge the presence of additional factors triggering ED use in elderly. Nevertheless, our findings suggest a considerable share of ED resources attributable to ACSCs which can be successfully avoided with appropriate community and residential care. More importantly, the increase in ACSC-related ED use is intensifying. It is imperative that current numbers of preventable ED visits sought by older adults are reduced in order to alleviate strain on the already-overused health care system and front-line staff. This can be achieved via efficient monitoring and reinforcing of the quality of care in the NHs and community care.

## Conclusions

Here we examined the ACSC-related ED visits and found that, among the six ACSCs studied, pneumonia, UTI and sepsis were the leading causes of ED visits, subsequent hospital admissions, related mortality, health care costs and DALYs in Taiwanese elderly adults. We also demonstrated the significant difference in the prevalence and the odds of ACSC-attributable ED care between NH and non-NH residents. Findings from this study in Taiwan can be implied for other countries with similar aging demographics where rising health care spending and unnecessary health care resource utilization pose a great public health issue.

## Supplementary information

Supplementary information

## Data Availability

All data generated or analysed in this study are included in this published article (and its Supplementary Information files).
