# Supplementary material for: Emergency Department Visits and Disease Burden Attributable to Ambulatory Care Sensitive Conditions in Elderly Adults
Source: Sci Rep. 2019 Mar 7;9:3811. doi: 10.1038/s41598-019-40206-4 (PMC6405841; doi:10.1038/s41598-019-40206-4)
Supplement: Supplementary file 1 — Supplementary information [file 41598_2019_40206_MOESM1_ESM.docx]

**Supplementary Materials**

**Emergency Department Visits and Disease Burden Attributable to Ambulatory Care Sensitive Conditions in Elderly Adults**

Vivian Chia-Rong Hsieh,^1^ Meng-Lun Hsieh,^2^ Jen-Huai Chiang,^1,3^ Andy Chien,^4^ Ming-Shun Hsieh,*^5,6,7,8^

^1^ Department of Health Services Administration, China Medical University, Taichung, Taiwan;

^2^ School of Chinese Medicine, China Medical University, Taichung, Taiwan;

^3^ Management Office for Health Data, China Medical University Hospital, Taichung, Taiwan;

^4^ Department of Physical Therapy and Graduate Institute of Rehabilitation Science, China Medical University, Taichung, Taiwan;

^5^ Institute of Occupational Medicine and Industrial Hygiene, College of Public Health, National Taiwan University, Taipei, Taiwan;

^6^ Department of Emergency Medicine, Taipei Veterans General Hospital, Taoyuan Branch, Taoyuan, Taiwan;

^7^ Department of Emergency Medicine, Taipei Veterans General Hospital, Taipei, Taiwan;

^8^ School of Medicine, National Yang-Ming University, Taipei, Taiwan;

***Correspondence to:**

Ming-Shun Hsieh, MD

Department of Emergency Medicine,

Taipei Veterans General Hospital, Taoyuan Branch,

No. 100, Sec. 3, Cheng-Kung Road,

Taoyuan 330, Taiwan, R.O.C.

E-mail: edmingshun@gmail.com

46478.4

93107.6

136701.4

**Figure S1 Trend in overall emergency department visits among the elderly, 2002-2013**

**Table S1 Overall emergency department visits by patient and hospital attributes, 2002-2013**

| **Variable** |  | **Visits** | | | |
| --- | --- | --- | --- | --- | --- |
|  |  | **n** | **%** | **SE** | **95% CI** |
| Overall |  | 563,647 | 100 | -- | -- |
| Age (year) |  |  |  |  |  |
|  | 65-69 | 116,663 | 20.7 | 0.213 | (20.28-21.12) |
|  | 70-74 | 128,963 | 22.9 | 0.221 | (22.45-23.31) |
|  | 75-79 | 128,602 | 22.8 | 0.239 | (22.35-23.28) |
|  | 80-84 | 105,816 | 18.8 | 0.227 | (18.33-19.22) |
|  | >=85 | 83,603 | 14.8 | 0.159 | (14.52-15.14) |
| Sex |  |  |  |  |  |
|  | Male | 299,344 | 53.1 | 0.168 | (52.78-53.44) |
|  | Female | 264,303 | 46.9 | 0.162 | (46.57-47.21) |
| Area of residence |  |  |  |  |  |
|  | North | 249,132 | 44.2 | 0.177 | (43.85-44.55) |
|  | Center | 103,817 | 18.4 | 0.215 | (18-18.84) |
|  | South | 163,138 | 28.9 | 0.186 | (28.58-29.31) |
|  | East | 28,356 | 5.0 | 0.269 | (4.5-5.56) |
|  | Remote Islands | 5,616 | 1.0 | 0.253 | (0.5-1.49) |
| Facility level |  |  |  |  |  |
|  | Medical center | 177,774 | 31.5 | -- | (31.54-31.54) |
|  | Regional hospital | 244,410 | 43.4 | -- | (43.36-43.36) |
|  | District hospital | 135,045 | 24.0 | -- | (23.96-23.96) |
|  | Local clinic | 2,350 | 0.40 | -- | (0.42-0.42) |
| Nursing home residents |  |  |  |  |  |
|  | No | 547,468 | 97.1 | 0.039 | (97.05-97.21) |
|  | Yes | 16,799 | 2.98 | 0.366 | (2.26-3.7) |

**Table S2 Hospital admissions and deaths subsequent to emergency department visits stratified by age group, 2002-2013**

| **Age group** | **All** |  | **65-69y** |  | **70-74y** |  | **75-79y** |  | **80-84y** |  | **>=85y** |  |
| --- | --- | --- | --- | --- | --- | --- | --- | --- | --- | --- | --- | --- |
|  | **n** | **%** | **n** | **%** | **n** | **%** | **n** | **%** | **n** | **%** | **n** | **%** |
| ED visits | 563,647 | 100 | 116,663 | 100 | 128,963 | 100 | 128,602 | 100 | 105,816 | 100 | 83,603 | 100 |
| Hospital admissions | 66,259 | 11.8 | 10,980 | 9.4 | 13,651 | 10.6 | 14,798 | 11.5 | 13,460 | 12.7 | 13,370 | 16.0 |
| Deaths | 13,583 | 2.4 | 2,814 | 2.4 | 2,870 | 2.2 | 3,378 | 2.6 | 2,629 | 2.5 | 1,892 | 2.3 |

ED: emergency department
